# COVID-19, gram-negative sepsis and a pleuro-peritoneal leak

**DOI:** 10.11604/pamj.2020.37.21.25311

**Published:** 2020-09-05

**Authors:** Poobalan Naidoo, Yeshkhir Naidoo

**Affiliations:** 1 Department of Nephrology, Inkosi Albert Luthuli Central Hospital, Durban, KwaZulu-Natal, South Africa,; 2 Department of Radiology, Inkosi Albert Luthuli Central Hospital, Durban, KwaZulu-Natal, South Africa

**Keywords:** COVID-19, pleuro-peritoneal leak, Acinetobacter baumannii

## Image in medicine

A 28-year old male with end stage renal disease, on continuous ambulatory peritoneal dialysis, presented with severe chest pain and dyspnoea at rest. Chest X-ray showed bilateral pleural effusions and when tapped had a high glucose concentration, suggesting a pleuro-peritoneal leak. Bilateral pleuro-peritoneal leaks were confirmed on nuclear medicine imaging. While awaiting pleurodesis he had another episode of severe chest pain. Chest X-ray showed a right sided pleural effusion with a vague opacity in the left upper zone. The pain resolved after parenteral opiate therapy and an acute coronary syndrome and pulmonary embolism were excluded. However, 18 hours later, he developed respiratory distress with type 1 respiratory failure. A repeat chest X-ray showed a circular opacification in the left upper zone. He was intubated, ventilated and a right sided chest drain inserted. He tested SARS-CoV-2 positive, and blood culture grew *Acinetobacter baumannii*. He demised post cardiopulmonary arrest.

**Figure 1:**
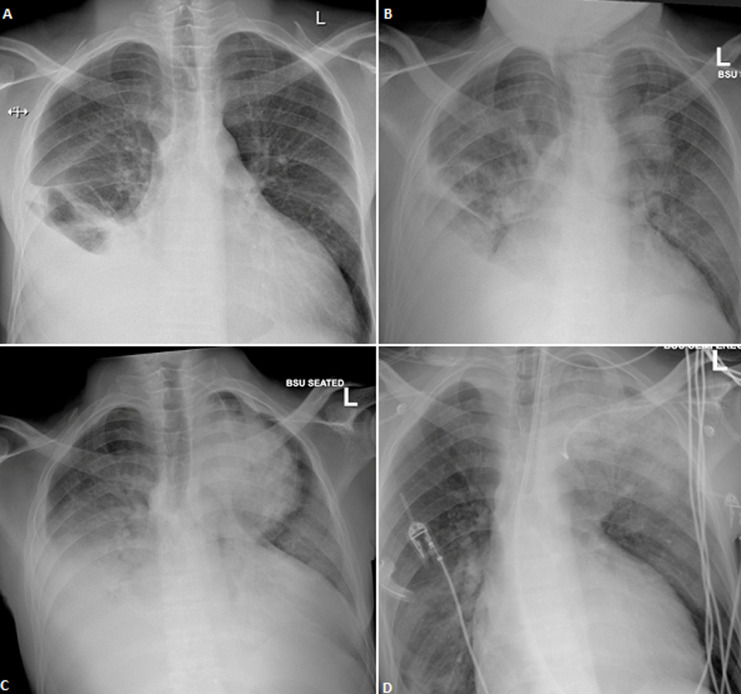
A) right sided pleural effusion; B) right sided pleural effusion with vague opacification in the left upper zone; C) right sided pleural effusion with circular opacification in the left upper zone; D) right sided intercostal chest drain tube with left upper zone consolidation

